# Obesity, diabetes, and other metabolic disorders among the Bororo Indigenous population

**DOI:** 10.20945/2359-4292-2026-0075

**Published:** 2026-07-03

**Authors:** Luiz F. Viola, Amaury Lelis Dal Fabbro, João Paulo Botelho Vieira-Filho, Laércio Joel Franco, Regina S. Moises

**Affiliations:** 1 Divisão de Endocrinologia, Escola Paulista de Medicina, Universidade Federal de São Paulo, São Paulo, SP, Brasil; 2 Departamento de Medicina Social, Faculdade de Medicina de Ribeirão Preto, Universidade de São Paulo, São Paulo, SP, Brasil

**Keywords:** Indigenous population, weight excess, Brazil, metabolic disorders

## Abstract

**Objective:**

Obesity and diabetes are widely recognized risk factors for cardiovascular disease, and their increase among Indigenous populations has been documented in the medical literature. Given the regional differences in the prevalence of metabolic disorders, this study aimed to evaluate the prevalence of obesity, diabetes, and other metabolic conditions in the Bororo population of the Central-West region of Brazil.

**Subjects and methods:**

In this cross-sectional study, 152 Bororo individuals from the Meruri Reservation in Mato Grosso, Brazil, underwent clinical, anthropometric, and laboratory assessments.

**Results:**

Women presented a worse metabolic profile than men, demonstrating significantly higher body mass index, waist circumference, total cholesterol, LDL-c, and 2-hour glucose levels, whereas men exhibited higher systolic blood pressure. Obesity was observed in 30.2% of the participants, with a higher prevalence among women (40.0% vs. 21.9%, p = 0.02). Prediabetes affected 51.3% of the participants, showing a higher prevalence in older men than in younger men (70.6% vs. 43.7%, p = 0.02). Diabetes was diagnosed in 9.2% of the participants, exclusively among women (20.0%), and rose to 38.7% in women aged ≥40 years. Hypertension was present in 23.0% of the participants and was positively associated with age. Central obesity was highly prevalent in this study cohort (72.3%).

**Conclusion:**

Our findings revealed an unfavorable metabolic profile among the Bororo Indigenous population, particularly in older women. This underscores the critical need for sexand age-specific preventive measures and management strategies for metabolic diseases in this population.

## INTRODUCTION

Indigenous Brazilian populations have experienced significant lifestyle changes driven by territorial and cultural losses. The depletion of natural resources and increasing urbanization have marginalized traditional practices, such as fishing and hunting, while concurrently increasing exposure to ultra-processed foods (^1^,^2^). This transition has profoundly altered dietary and physical activity habits, which act as key determinants of health (^2^,^3^).

While infectious diseases remain a significant cause of morbidity among Indigenous people (^4^), the high prevalence of metabolic disorders has exacerbated health inequities between Indigenous and non-Indigenous individuals (^2^,^5^). The Bororo is an indigenous Brazilian population inhabiting six demarcated territories within the state of Mato Grosso. These territories are currently 300 times smaller than their traditional lands and have been severely degraded by deforestation and mining activities (^6^). The Meruri Reservation, one of the largest Bororo territories, supports an estimated population of 811 individuals, 47% of whom are under 17 years old (^7^). The Xavante, another Indigenous population inhabiting the same geographic region, differ in their ethnic identity and certain cultural practices. Previous studies have shown a high prevalence of diabetes (28.2%) and obesity (50.8%) within this population (^8^,^9^). Given this context, we aimed to investigate the prevalence of metabolic disorders, including prediabetes, diabetes, obesity, and hypertension, among the Bororo.

## SUBJECTS AND METHODS

This cross-sectional study was conducted among the Bororo Indigenous population living in the Meruri Reservation. All individuals aged ≥17 years, excluding pregnant women, were invited to participate. Demographic data, medical history, and current medication use were recorded. Body weight was measured using a digital scale (Plenna), with participants wearing light clothing. Height was measured using a stadiometer (AlturExata). Body mass index (BMI) status was categorized according to World Health Organization standards (^10^). Waist circumference (WC) was considered elevated based on established cut-off points for South American populations: ≥90 cm for men and ≥80 cm for women (^11^).

Blood pressure (BP) was assessed using an automatic sphygmomanometer (OMRON HEM-742INTC^®^). Three sequential readings were taken, and the average of the last two was used for analysis. Hypertension was defined as a systolic BP of ≥140 mmHg, a diastolic BP of ≥90 mmHg, or the active use of antihypertensive medication (^12^). An oral glucose tolerance test (OGTT) was administered to participants with an initial fasting glucose (FG) of <200 mg/dL who were not taking antidiabetic medications. Capillary glucose levels were measured using a portable glucometer (HemoCue^®^). Diabetes was diagnosed based on the current use of antidiabetic drugs, a fasting or 2-hour post-glucose load capillary glucose level of ≥200 mg/dL, or an HbA1c level of ≥6.5%. Prediabetes was defined as having a 2-hour glucose level between 140 and 199 mg/dL or an HbA1c level of 5.7-6.4%. Cholesterol, HDL-c, and triglycerides were measured enzymatically. LDL-c was calculated using the Friedewald formula. HbA1c testing was performed via high-performance liquid chromatography (Tosoh G7, USA).

The normality of all continuous variables was assessed using the Shapiro-Wilk test (5% significance level). Data are presented as the mean ± standard deviation (SD) for continuous variables and as frequencies (categorical). Group comparisons were performed using Student’s t-test, the Mann-Whitney U test, and Fisher’s exact test, as appropriate. Statistical analyses were conducted using Stata/BE version 18.5 (StataCorp LLC, USA). The study protocol was approved by the Ethics Committee of the Escola Paulista de Medicina, Universidade Federal de São Paulo, and the Brazilian National Ethics Committee (approval no. 7.566.271, CAAE no. 79870324.3.0000.5505).

## RESULTS

The study enrolled 152 participants (82 men and 70 women), with a mean age of 39.7 ± 14.6 years. **Table 1** summarizes their clinical characteristics by sex. Women exhibited significantly higher values for BMI, WC, total cholesterol, LDL-c, and 2-hour post-load glucose, whereas men demonstrated higher systolic BP. Age-stratified analyses (<40 years vs. ≥40 years) indicated that aging exerted more pronounced adverse metabolic effects on women than on men (**Table 2**). Older women had substantially higher rates of obesity, hypertension, and diabetes than their younger counterparts. Overall, diabetes was identified in 9.2% of the participants, exclusively in women, affecting 20% of them and 38.7% of women aged ≥40 years. Among individuals diagnosed with diabetes, the mean HbA1c was 9.8% ± 3.1% (range: 6.0%-14.8%). Five individuals (35.0%) had an HbA1c of ≤7.0%, two (14.0%) had levels between >7.0% and <8.0%, and seven (50.0%) exhibited levels ≥8.0%.

**Table 1:** Demographic, anthropometric, and metabolic variables of the study population according to sex

Variable	Men n = 82 (53.9%)	Women n = 70 (46.1%)	Total n = 152	*p*
Age (years)	38.9 ± 15.4	40.5 ± 13.6	39.7 ± 14.6	0.38
BMI (kg/m^2^)	27.1 ± 4.4	29.2 ± 5.7	28.1 ± 5.1	0.03
WC (cm)	91.3 ± 11.7	97.5 ± 11.9	94.2 ± 12.1	<0.01
Basal glucose (mg/dL)	107.3 ± 13.7	120.2 ± 50.5	113.3 ± 36.1	0.68
2-h 75-g OGTT (mg/dL)	135.1 ± 22.2	152 ± 39.3	142.4 ± 31.8	0.01
Total cholesterol (mg/dL)	138.3 ± 30.1	154.3 ± 34.4	145.7 ± 33	<0.01
LDL-c (mg/dL)	70.2 ± 24.0	81.1 ± 29.3	75.2 ± 27	0.01
HDL-c (mg/dL)	47.9 ± 16.8	50.9 ± 16.5	49.3 ± 16.7	0.09
Triglycerides (mg/dL)	108.2 ± 60.4	123.1 ± 75.2	115.1 ± 67.8	0.12
Systolic BP (mmHg)	120.7 ± 17.3	112.5 ± 19.8	116.9 ± 18.9	<0.01
Diastolic BP (mmHg)	70.3 ± 12.5	68.8 ± 10.4	69.6 ± 11.6	0.56

Abbreviations: BMI: body mass index; WC: waist circumference; OGTT: oral glucose tolerance test; LDL-c: low-density lipoprotein cholesterol; HDL-c: high-density lipoprotein cholesterol; BP: blood pressure; systolic BP: systolic blood pressure; diastolic BP: diastolic blood pressure.

**Table 2:** Metabolic profile by sex and age groups in the study population

Variable	Women (n = 70)					Men (n = 82)					Total (n = 152)	
Age	< 40y (n = 39)	≥ 40y (n = 31)	^*^ *p*	All women		< 40 y (n = 48)	≥ 40 y (n = 34)	^*^ ***p***	All men		Total	^#^ *p*
Increased WC	36 (92.3%)	31 (100%)	0.24	67 (95.7%)		22 (45.8%)	21 (61.8%)	0.18	43 (52.4%)		110 (72.3%)	<0.01
Overweight	18 (46.1%)	7 (22.6%)	0.04	25 (35.7%)		20 (41.6%)	16 (47.1%)	0.65	36 (43.9%)		61 (40.1%)	0.32
Obesity	11 (28.2%)	17 (54.8%)	0.02	28 (40%)		09 (18.8%)	09 (26.5%)	0.42	18 (22%)		46 (30.2%)	0.02
Hypertension	4 (10.3%)	18 (58.1%)	<0.01	18 (25.7%)		05 (10.4%)	12 (35.2%)	0.01	17 (20.7%)		35 (23%)	0.56
Diabetes mellitus	02 (5.1%)	12 (38.7)	<0.01	14 (20%)		0	0	-	0		14 (9.2%)	<0.01
Prediabetes	16 (41%)	17 (54.8%)	0.33	43 (61.4%)		21 (43.7%)	24 (70.6%)	0.02	45 (54.9%)		78 (51.3%)	0.51

* *p*-values for comparison between age groups. #*p*-values for comparison between men and women. WC: waist circumference.

Prediabetes was identified in 51.3% of the participants, with no significant differences overall between the sexes. However, among men, prediabetes affected 54.9% and was notably more prevalent in those aged ≥40 years compared to younger men (70.6% vs. 43.7%, *p* = 0.02). Hypertension affected 23.0% of the participants, with equal overall distribution between the sexes, though its prevalence increased sharply with age, affecting 58.1% of older women and 35.2% of older men.

Excess weight was highly prevalent, affecting 70.3% of all participants. Obesity affected 30.2% of the cohort and was significantly more common in women than in men (40.0% vs. 21.9%, *p* = 0.02). Obesity rates were much higher in older women than in younger women (54.8% vs. 28.2%, *p* = 0.02). Overweight status was observed in 40.1% of the individuals. Only 29.6% had a normal BMI, and no participants were classified as underweight. Central obesity (increased WC) was broadly identified, affecting 72.3% of participants. The prevalence was overwhelmingly higher in women than in men (95.7% vs. 52.4%, *p* < 0.001), with 100% of women aged ≥40 years meeting the threshold criteria. Notably, even among women with normal weight (n=17), 88.0% (n = 15) presented with central obesity.

## DISCUSSION

The Bororo exhibit higher rates of obesity (30.2%) and overweight (40.1%) compared to the general Brazilian adult population (25.9% and 34.4%, respectively) (^13^). Previous meta-analyses of Brazilian Indigenous adults from various ethnic groups reported lower overall rates of obesity (18%) and overweight (33%) (^2^). However, a systematic review of adult Indigenous populations in Brazil identified peak obesity prevalence among the Guarani, Kaiowá, Khisedje, and Terena groups (31%), closely aligning with our findings for the Bororo (^14^). In our previous study characterizing the Xavante population, obesity rates were even higher, affecting 52.4% of women and 47.6% of men (^15^). The Xavante and the Bororo inhabit the same geographic region and share similar patterns of interaction with the non-Indigenous population. The higher obesity rates observed in the Xavante may be attributable to their remarkably low genetic admixture (^16^) combined with a rapid nutritional acculturation toward a Westernized diet. While the Xavante have engaged in contact with non-Indigenous society primarily since the 1940s, formal contact for the Bororo dates back much further, to 1902 (^17^). This rapid nutritional acculturation may have driven the elevated obesity rates among the Xavante.

Sex disparities regarding obesity were prominent in our study; women demonstrated higher rates than men (40.0% vs. 21.9%), mirroring established patterns in both the general Brazilian population (30.2% vs. 22.8%) (^13^) and other Indigenous groups (^15^,^18^). Waist circumference, a well-validated marker of visceral fat, is independently associated with cardiovascular risk across various populations (^19^,^20^). Central obesity was notably high among Bororo women, with all women aged >40 years, showing increased waist circumference, indicating substantial cardiometabolic risk.

The overall prevalence of diabetes in the Bororo population (9.2%) was comparable to that of the general Brazilian adult population, which ranges from 6.6% to 9.4% depending on the diagnostic criteria employed (^21^). Among the Bororo, however, diabetes was diagnosed exclusively in women, particularly those aged ≥40 years. Pronounced sex discrepancies in diabetes rates among Indigenous populations are well documented, with a predictably higher prevalence in women across various Indigenous groups (^22^,^23^). However, on a global scale, the prevalence of diabetes is generally higher in men or remains comparable between sexes (^22^,^24^). The overwhelming prevalence of obesity, notably central adiposity, among Bororo women likely contributes substantially to these escalated diabetes rates. Factors such as relative differences in physical activity, early menarche, previous instances of gestational diabetes, high parity, unfavorable body composition changes, increased postmenopausal insulin resistance, and various psychosocial determinants, may conjointly explain the greater diabetes burden among Bororo women.

Prediabetes affected 51.3% of the total sample, a figure substantially higher than any rate normally reported for the general Brazilian population. For context, Malta and cols. (^21^) found a 16.9% prevalence using the HbA1c criterion, whereas Schmidt and cols. (^25^) reported a 20.3% prevalence utilizing the oral glucose tolerance test. Although prediabetes is a firmly established risk factor for both diabetes and cardiovascular disease (^26^), only Bororo women developed diabetes, despite similar prediabetes rates, suggesting sex-specific factors. Higher obesity rates alongside variations in physical activity, hormonal profiles, and diet may increase women’s vulnerability to diabetes.

Hypertension was identified in 23.0% of participants, increasing with age. Among women, no cases were detected in those under 40 years of age, while 58.1% of those aged ≥40 years were hypertensive. Among men, the prevalence surged from 10.4% in the younger subset to 35.2% in those aged ≥40 years. In aggregate, these rates remain lower than the 30% rate observed in the Brazilian population (^27^), and are comparable to historical data derived from the Xavante population (^9^). Kramer and cols.’s review evaluating 46 studies of Indigenous Brazilian adults reported 11% overall hypertension prevalence, ranging from 1% in less urbanized regions to 30% in urban regions. This study noted that BP generally escalated alongside age specifically in urbanized Indigenous populations, although not in those maintaining traditional lifestyles (^2^). Furthermore, Oliveira and cols. (^28^) found a 29.5% prevalence of hypertension in an Indigenous community in Central Brazil. Ultimately, these collective findings highlight the volatile and highly varied prevalence of hypertension among Indigenous populations, differing by age, sex, region, and acculturation.

This study has several limitations. The characteristically irregular meal patterns typical of the Bororo people may have affected the accuracy of our fasting blood measurements. However, the 2-hour OGTT provides a more reliable assessment of glucose metabolism than FG alone, proving superior in predicting heart disease risk (^29^,^30^). Therefore, isolated impaired FG levels were not independently used to define instances of prediabetes. Triglyceride measurements were assumed to be in the fasting state, potentially introducing errors.

Our findings showed an unfavorable metabolic profile among the Bororo Indigenous population across numerous sex and age demographics. Women aged ≥ 40 years showed higher rates of obesity, especially central obesity, than men. Diabetes only occurred only in women, despite similar prediabetes rates between the sexes. These findings indicate the need for sexand age-specific strategies for treating metabolic diseases in these populations.

## Data Availability

the datasets used and/or analyzed during the current study are available from the corresponding author upon reasonable request.
